# Supplementary material for: Setting Goals to Reduce Cardiovascular Risk: A Retrospective Chart Review of a Pharmacist-Led Initiative in the Workplace
Source: Int J Environ Res Public Health. 2023 Jan 2;20(1):846. doi: 10.3390/ijerph20010846 (PMC9820010; doi:10.3390/ijerph20010846)
Supplement: Supplementary file 1 [file ijerph-20-00846-s001.zip › ijerph-2022714-supplementary.pdf]

International Journal of
Environmental Research and Public Health
MDPI

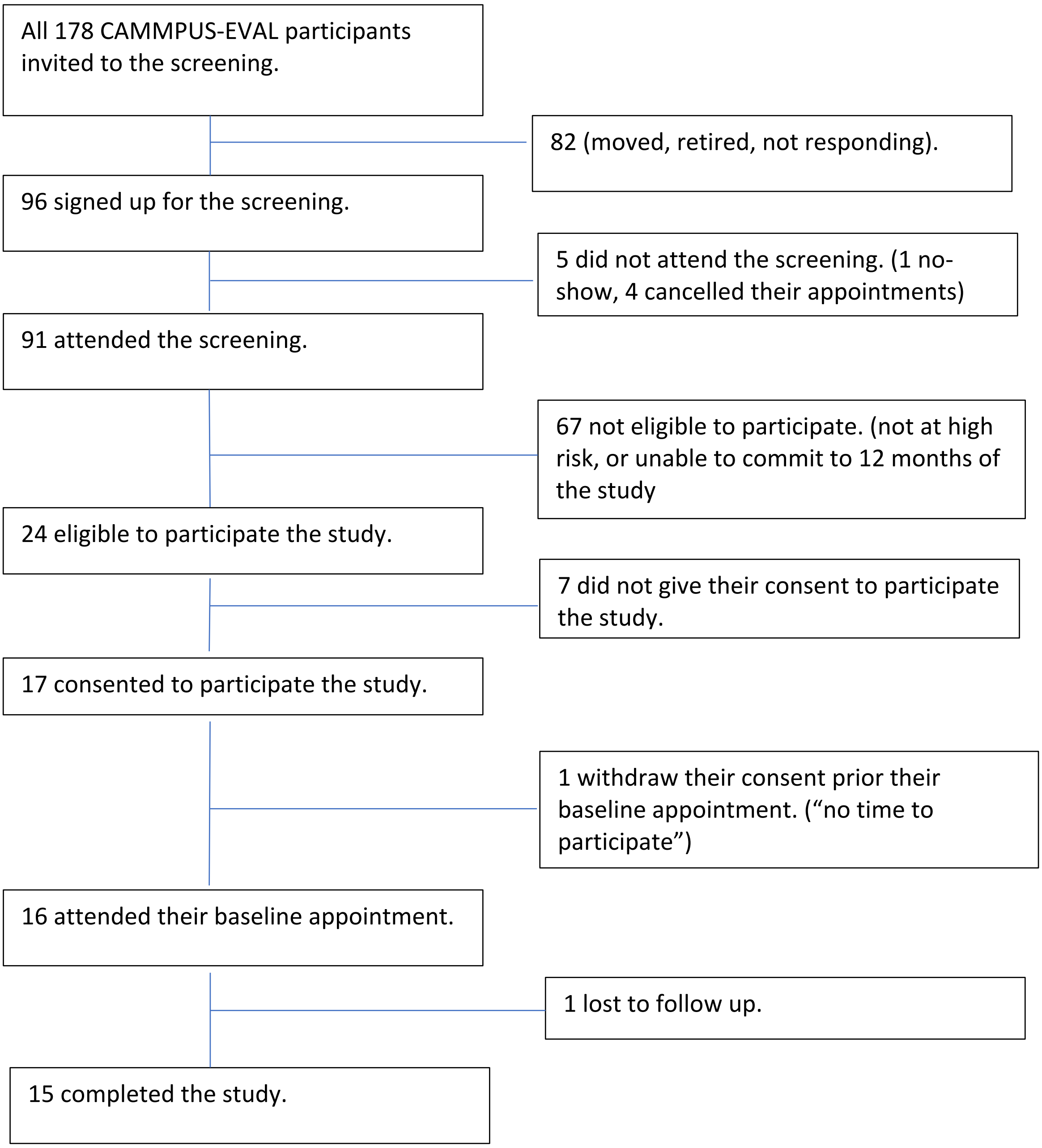

Supplementary Figure S1. Flow chart of patient selection and study sample size.

Supplementary Table S1. CAMMPUS 2.0 Goal-Setting Questions.

Provider Questions
1. What is the general domain that the participant wants to work on?
2. What exactly are you going to do?
3. When exactly are you going to do it?
4. How often are you going to do this?
5. Where will you do this?
6. Are there any challenges or barriers that you anticipate?
7. How confident are you in achieving this goal? (from 0 - not at all, to 10 - completely)
8. When do you plan to start this action plan (if too far away, ask what they feel ready to start now)
